# Could the SARS-CoV-2 infection be acquired from Smartphones?

**DOI:** 10.4314/ejhs.v30i5.26

**Published:** 2020-09

**Authors:** Siukan Law, Ali Hassan A Alnasser, Jaffar A Al-Tawfiq

**Affiliations:** 1 Department of Science, School of Science and Technology, The Open University of Hong Kong, Ho Man Tin, Kowloon, Hong Kong; 2 Laboratory Department, Dhahran Eye Specialist Hospital, Ministry of Health, Eastern Province, Dhahran, Saudi Arabia; 3 Infectious Disease Unit, Specialty Internal Medicine, Johns Hopkins Aramco Healthcare, Dhahran, Saudi Arabia; 4 Department of Medicine, Indiana University School of Medicine, Indianapolis, IN, USA; 5 Department of Medicine, Johns Hopkins University School of Medicine, Baltimore, MD, USA

Dear Editor,

Severe acute respiratory syndrome coronavirus 2 (SARS-CoV-2) was identified in December 2019 and termed as coronavirus disease 2019 (COVID-19). It's highly contagious and linked to the human-to-human transmission. The World Health Organization (WHO) declared the COVID-19 outbreak on 30 January 2020 and announced it as a global pandemic on 11 March (1).

Rethinking to the modes of transmission on COVID-19 infection, SARS-CoV-2 might be transmitted by respiratory droplets within a long distance of 2m or by contaminated surfaces leading to infection through contact transmission (2). Respiratory etiquette and maintaining physical distances are key interventions to prevent such transmission of infection by respiratory droplets (3). However, can we avoid touching infected surfaces e.g., smartphones' touch screens? The SARS-CoV-2 symptomatic or asymptomatic patient who coughs or sneezes nearby any smartphones, then any individual who touches the smartphones might be inadvertently infected with the SARS-CoV-2. This may occur by touching the phone then touching the mouth, nose, and eyes leading to respiratory infection (4).

SARS-CoV and MERS-CoV have similar structures to the SARS-CoV-2 as well as the mode of transmission. SARS-CoV-2 remains on surfaces, including the plastered wall, formica, plastic, stainless steel, glass and SARS-CoV-2 remains infectious at room temperature for up to nine days (5). Since SARS-CoV-2 is suitable to survive at low temperatures and high humidity conditions, its lifespan also would be enhanced (6). In 2015, Foong YC, et al. indicated that smartphones are potential vehicles for pathogenic and non-pathogenic bacteria in the hospital setting (7). Research indicated that the rate of isolation of non-pathogenic organisms were as follows: Coagulasenegative Staphylococci (58.8%), Bacillus spp. (6.2%), Diphtheroid spp. (11.5%) and Non-Hemolytic Streptococcus (10.6%) while there is only a 5% rate of contamination with potentially pathogenic organisms such as *Staphylococcus aureus* and *coliforms* (3). Handwashing with 80% ethanol or 75% 2- propanol recommended by the World Health Organization (WHO) inactivates viruses and bacteria (8). Alcohol inactivates enveloped viruses via denaturing proteins and has a veridical effect on SARS-CoV and MERS-CoV. The use of 0.1% sodium hypochlorite or 62–71% ethanol for surface disinfection also reduces the SARS-CoV-2 transmission (9).

Based on the provided information, smartphone devices can be a mediator in the transmission of infectious diseases, including SARS-CoV-2 in healthcare centers and the community. It is known that smartphones are no longer only for phone calls, but their use is necessary for communication, health information, e-learning, and medical consultations. Strategies should go beyond the imposition of behavioral controls for individuals with a commitment to regularly disinfect smartphones, portable electronic medical record devices, etc. Besides, finding alternative ways to use these devices in a clinical setting is paramount importance.
