# Supplementary material for: Could the SARS-CoV-2 infection be acquired from Smartphones?
Source: Ethiop J Health Sci. 2020 Sep;30(5):853–4. doi: 10.4314/ejhs.v30i5.26 (PMC8047268; doi:10.4314/ejhs.v30i5.26)
Supplement: Related texts

# Reply: Response from The Editor

Thank you very much for raising this important issue on current condition. Having read the arguments and the evidences you cited and other sources, I concure with you that smart phone devices could be mediator of Corona virus like any other utensil and objects do. That is why sanitizing them is taken as one of the COVID-19 transmission preventing measures.
